# The Platelet to Lymphocyte and Neutrophil to Lymphocyte Ratios in Predicting Response to Platinum-based Chemotherapy for Epithelial Ovarian Cancer

**DOI:** 10.31557/APJCP.2021.22.5.1561

**Published:** 2021-05

**Authors:** Gatot Nyarumenteng Adhipurnawan Winarno, Marihot Pasaribu, Herman Susanto, Aisyah Shofiatun Nisa, Ali Budi Harsono, Hariadi Yuseran, Dodi Suardi, Nurvita Trianasari

**Affiliations:** 1 *Department of Obstetrics and Gynecology, Faculty of Medicine, Universitas Padjadjaran/Hasan Sadikin Hospital, Bandung, Indonesia. *; 2 *Department of Obstetrics and Gynaecology Medical Faculty Universitas Mulawarman/Abdul Wahab Sjahranie Hospital, Samarinda, Indonesia. *; 3 *Department of Obstetrics and Gynaecology Medical Faculty Universitas Mulawarman/Ulin Hospital, Banjarmasin, Indonesia. *; 4 *Telkom of Economics and Business School, Telkom University, Bandung, Indonesia. *

**Keywords:** Platelets/lymphocytes ratio, neutrophil/lymphocytes ratio, platinum-based chemotherapy

## Abstract

**Objective:**

The patients with advanced-stage ovarian cancer have higher factors complicating surgery; thus, the best choice for them is surgery with chemotherapy with six cycles of adjuvant chemotherapy. Generally, chemotherapy can be evaluated in various ways, phsychal examination, radiology examination, and laboratory examination. This study aims is to examine if the measurement of the platelet/lymphocyte ratio (PLR) and neutrophil/lymphocyte ratio (NLR) can be used to predict a patient’s response to chemotherapy.

**Methods:**

Analytic observational study with a case-control design conducted in the Dr. Hasan Sadikin Hospital in Bandung from 2017 to 2018. This study used the medical record of ovarian cancer patients with post-surgery complete blood counts and histopathological reports. The sample size was determined based on the categorical test’s statistical calculation to obtain a total number of at minimal 90 samples. All the study subjects who had undergone complete chemotherapy were followed up for 6 months. Their response to chemotherapy was assessed with a clinical examination, ultrasonography, and a CA-125 blood test every 3 months.

**Results:**

In 2017–2018, 504 patients were diagnosed with ovarian cancer at the Dr. Hasan Sadikin Hospital in Bandung, Indonesia. After reassessment, 116 patients had stage I to III ovarian cancer and underwent cytoreduction followed by platinum chemotherapy. The age, cancer stage, and types of epithelial cells in the platinum-sensitive and platinum-resistant patients were characterized. There were significant differences between the two groups in age and cancer stage characteristics (p < 0.05). The increase in platelet/lymphocyte (p = 0.003) and neutrophil/lymphocyte ratios (p = 0.026) are associated with the increase in the response to platinum chemotherapy against epithelium-based cancers.

**Conclusion:**

A patient’s NLR and PLR are strongly associated with his response to chemotherapy.

## Introduction

Ovarian cancer, the 8th most common cancer in a woman, has increased by more than 20% since 2012 globally (Sung et al., 2021). In 2018, the number of new ovarian cancer cases exceeded 300,000. In Indonesia, ovarian cancer is the third leading cause of cancer death in women after breast and cervical cancer (Indonesia, 2019). About 80%–90% of the ovarian malignancies are due to epithelial differentiation, with 19.6%–35% incidence (Zhang et al., 2019).

Cytoreduction surgery is the primary treatment for ovarian carcinoma. Patients with early-stage ovarian cancer are most suitable for cytoreduction. On the other hand, the patients with advanced-stage ovarian cancer have higher factors complicating surgery; thus, the best option is surgery with chemotherapy with six cycles of adjuvant chemotherapy (Querleu et al., 2017). Chemotherapy with platinum/carboplatin combined with paclitaxel is still the standard chemotherapy in ovarian carcinoma in Hasan Sadikin Hospital Bandung.

During chemotherapy, the inflammatory process plays a critical role in tumor cells’ sensitivity to chemotherapy (Savant et al., 2018). The progressive characteristics of ovarian cancer, which plays a role in tumor progression and metastasis and predicting the therapeutic effect of the chemotherapy and the prognosis of ovarian cancer, are influenced by the tumor microenvironment (TME)(Edwardson et al., 2017; Savant et al., 2018).

The tumor microenvironment’s activity is indicated by systemic inflammatory markers such as platelets, lymphocytes, and neutrophils (Savant et al., 2018). Apart from contributing to thrombus formation, platelets also play a role in tumor progression (Menter et al., 2014; Schlesinger, 2018). Platelet production is increased in cancer patients (Olsson and Cedervall, 2018). Vascular susceptibility contributes to platelet activation due to exposure to subendothelial factors such as tissue factors and collagen (Olsson and Cedervall, 2018). Tissue factors and cancer procoagulants produced by tumors and TMEs also contribute to platelet activation (Menter et al., 2014). A high platelet count and a high platelet-to-lymphocyte (platelet/lymphocyte) ratio are known to be indicators of poor prognostic in many types of cancer. While the high PLR is associated with low patient survival rate, a high lymphocyte-to-monocyte ratio is associated with a better colorectal cancer (CRC) outcome (Guo et al., 2017).

Besides platelet, neutrophils affect the immune system to facilitate tumor proliferation. Neutrophils repress the response of tumor-suppressing CD8 + T lymphocytes by releasing nitric oxide synthase (iNOS) or arginase 1 (ARG1) upon TGFβ stimulation (Masucci et al., 2019). This process also produces matrix metalloproteinase 9 (MMP9), which plays an essential role in tumor initiation (Liu et al., 2015). Furthermore, tumor proliferation is mediated by the degradation of insulin receptor substrate 1 (IRS1) by neutrophil elastase and the activation of PI3K signaling (DeStefano and Jacinto, 2013; Geng et al., 2014; Martini et al., 2014). Ultimately, neutrophils will promote metastasis by inhibiting NK cell function and facilitating the extravasation of tumor cells (Coffelt et al., 2016; Ocana et al., 2017; Masucci et al., 2019).

Generally, chemotherapy can be evaluated in various ways, including imaging tests like CT scan, MRI, and USG, and blood tests like the CA-125 test. The measurement of the platelet/lymphocyte ratio (PLR) and neutrophil-to-lymphocyte (neutrophil/lymphocyte) ratio (NLR) is an easy and economical examination that all the hospitals can employ. Therefore, this study aimed to determine whether there was a relationship between PLR and NLR and response to chemotherapy in epithelial ovarian cancer.

## Materials and Methods

An observational study with a comparative study design was conducted in the Dr. Hasan Sadikin Hospital in Bandung, Indonesia, in 2017–2018. This study used the medical record of ovarian cancer patients, which documented the patients’ complete blood counts after surgery and contained histopathological reports.

*Patients*

The patient sample size was determined based on the categorical test’s statistical calculation to obtain a total minimum of 90 samples. The research subjects were patients who met the inclusion and exclusion criteria. The inclusion criteria were patients with epithelial ovarian cancer that underwent platinum chemotherapy after cytoreduction surgery and did not suffer from chronic infectious diseases, bone marrow, blood disorders, and immunosuppression. Meanwhile, the exclusion criteria were the patients with incomplete blood tests and histological examinations.

*Blood tests*

All the study subjects who had undergone complete chemotherapy were followed up for 6 months. Their response to chemotherapy was assessed with a clinical examination, ultrasonography, and a CA-125 blood test every 3 months. The patients with deterioration or a persistent tumor mass fewer than 6 months after chemotherapy were classified as platinum-resistant. Meanwhile, the platinum-sensitive patients were patients with no deterioration or permanent tumor mass more than 6 months after chemotherapy. All the complete blood count data were obtained after cytoreduction surgery.

*Statistical analysis*

The statistical analysis was performed using SPSS version 24.0 for Windows to conduct the normality tests (the Kolmogorov-Smirnov tests), significance tests (unpaired t-test and Mann-Whitney U test), and categorical tests (Chi-Square).

## Results

In 2017–2018, 504 patients were diagnosed with ovarian cancer at the Dr. Hasan Sadikin Hospital in Bandung, Indonesia. After reassessment, 116 patients had stage I to III ovarian cancer and underwent cytoreduction followed by platinum chemotherapy, and the rest were advanced-stage patients, refuse surgery and or chemotherapy. The age, cancer stage, and types of epithelial cells in the platinum-sensitive and platinum-resistant patients were characterized (Table 1). There were significant differences between the two groups in age and cancer stage characteristics (p < 0.05). The mean age of patients in the platinum-sensitive group was 43.29 + 11.090, while the platinum-resistant group was 53.04 + 9.402.

Also, the PLR and NLR of the platinum-sensitive and platinum-resistant patients were compared (Table 2). The platinum-sensitive and platinum-resistant patients had significantly different PLR and NLR values (p < 0.05). The platinum-sensitive group had a higher median value of PLR value, at 14906.30, than that of the resistant group at 9788. Similarly, the platinum-sensitive patients had a higher median NLR value, at 2.70, than the platinum-resistant group at 1.79. The use of PLR as a predictor factor for chemotherapy had a cut value of 11717.40 while that of NLR was 2.56 (Table 3).

**Figure 1:**
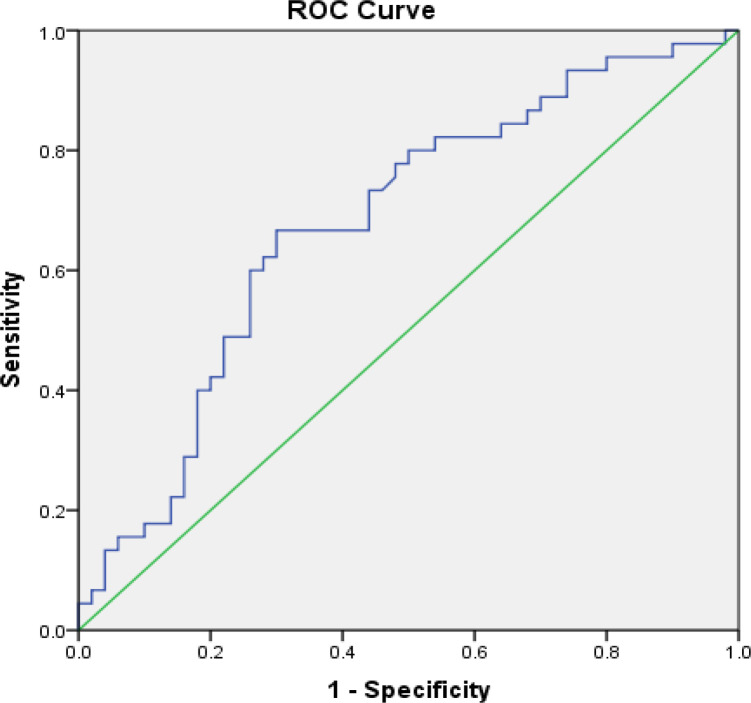
A Receiver Operating Characteristic (ROC) Curve of the Correlation of Platelet Lymphocytes Ratio (PLR) to Response to Chemotherapy

**Figure 2:**
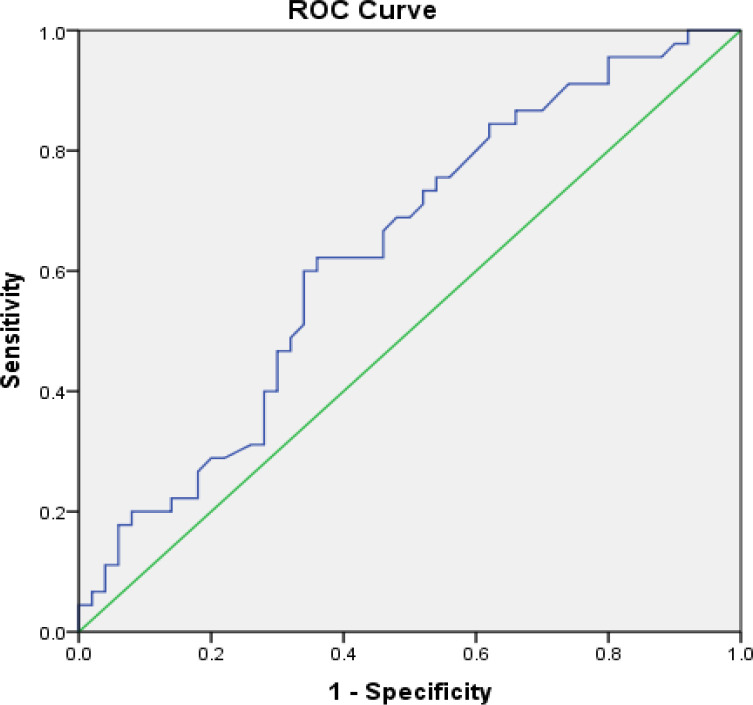
A Receiver Operating Characteristic (ROC) Curve of the Correlation of Neutrophil Lymphocytes Ratio (NLR) to Response to Chemotherapy

The PLR or neutrophil/lymphocyte ratio (NLR) among the platinum-sensitive and platinum-resistant patients were compared (Table 3) based on the cutoff point (COP), which was obtained from the Receiver Operating Characteristic (ROC) analysis (Figures 1 and 2). Based on the ROC curve, the optimum cutoff value for NLR was 2.56 with a sensitivity value of 60% and a specificity value of 66%. Based on the AUC value of 63.2%, the patients with an NLR value of more than 2.56 were prognostic factors for platinum-sensitive chemotherapy. In summary, the use of PLR as a predictive factor for chemotherapy success had the COP value of 11717.40 while NLR had the COP value of 2.56.

The ROC curves of PLR and NLR (Figures 1 and 2) as predictive factors for patients’ response to chemotherapy had the AUC value of 67.8% and 63.2%, respectively. Both curves had a good diagnostic value because they were away from the 50% line and close to 100%.

**Table 1:** The Characteristics of the Ovarian Cancer Patients who Underwent Chemotherapy

Variable	Group		P-Value
	Platinum- Sensitive n (%)	Platinum- Resistant n (%)	
Age (years)			0.0001
Mean ± STD	43.29 ± 11.090	53.04 ± 9.402	
Median	44:00:00	54:00:00	
Stage			0.039
I	19 (42.2)	15 (30.0)	
II	6 (13.3)	18 (36.0)	
III	20 (44.4)	17 (34.0)	
Cell type			0.208
Serous	9 (20.0)	20 (40.0)	
Mucinous	22 (48.9)	7 (14.0)	
Endometrioid	5 (11.1)	15 (30.0)	
Clear cell	7 (15.6)	7 (14.0)	

**Table 2:** The Platelets/Lymphocytes and Neutrophil/Lymphocyte Ratio in the Platinum-Sensitive and Platinum-Resistant Ovarian Cancer Patients

Variable	Group		P-Value
	Platinum-Sensitive n (%)	Platinum-Resistant n (%)	
Platelet/lymphocyte ratio			0.003
Mean ± STD	20073.36 ± 15101.14	13746.40 ± 12389.31	
Median	14906.30	9788:10:00	
Neutrophil/lymphocyte ratio			0.026
Mean ± STD	3.31 ± 2.653	2.37 ± 1.751	
Median	0.132	0.096	

**Table 3:** Comparing the Proportions of Platinum-Sensitive and Platinum-Resistant Patients above or below the Cutoff Value of the Platelet/Lymphocyte or Neutrophil/LymphocyteRatio

Variable	Group		P-value
	Platinum-sensitive	Platinum-resistant	
	n (%)	n (%)	
lymphocytes lymphocyte			0.0001**
>11717.40	30 (66.7)	15 (30.0)	
<11717.40	15 (33.3)	35 (70.0)	
Neutrophil/lymphocyte ratio			0.011 **
>2.56	27 (60.0)	17 (34.0)	
<2.56	18 (40.0)	33 (66.0)	

## Discussion

Ovarian malignancy can occur in any age group. Here the patients under the age of 50 were found to have a better response to chemotherapy (Table 1), as the platinum-sensitive patients had a lower average age than the platinum-resistant group (p < 0.05). A study conducted by Badora-Rybicka assessed 315 medical records of newly diagnosed ovarian cancer patients and found that the platinum-sensitive group’s mean age was lower than that of the platinum-resistant group (Badora-Rybicka et al., 2016). Also, Deng et al., (2017) stated that progression-free survival was closely related to the age cutoff of 65. In contrast, Cho et al., (2009) stated that age of 51 or older was a poor predictor of chemotherapy response. Apart from age, cancer stage was found to be associated with the response to platinum therapy (p < 0.05), (Mendiola et al., 2018) in line with Mendiola’s observation that the ovarian cancer stage affects chemotherapy response (Mendiola et al., 2018).

The types of epithelial cells found from ovarian cancer patients in this study were mostly serous and mucinous (Table 1); there were no differences in cell type between the platinum-sensitive and resistant groups (p < 0.030). These data are in line with the finding of Supachai Raungkaewmanee et al. that the type of ovarian cancer cells does not influence the prognosis of ovarian cancer (Raungkaewmanee et al., 2012).

Since PLR and NLR can be used to assess the progression of different types of cancers, such as non-small-cell lung cancer, hepatocellular carcinoma, nasopharyngeal carcinoma, colorectal melanoma, and breast cancer, (Azab et al., 2013; Neofytou et al., 2014; Zhou et al., 2014; Diem et al., 2017; Jiang et al., 2018; Zuo et al., 2019) their values were compared in the platinum-sensitive and platinum-resistant patients (Table 2). The platinum-sensitive group had higher median PLR and NLR values than the resistant group.

This study found that the greater PLR ratio was related to better response to chemotherapy. This study’s results are in line with research conducted by Badora-Rybicka, who also took samples before chemotherapy in 2016. That study found that the greater the PLR value was associated with better progression-free survival (Badora-Rybicka et al., 2016). A change in this ratio could be caused by various things, including changes in platelet and lymphocyte levels after surgery (Hu et al., 2020). In contrast, Raungkaewmanee et al. found that the PLR value over 200 was associated with more aggressive tumors and lower patient survival (Raungkaewmanee et al., 2012). The difference in the observations may be due to the methodology, namely the sampling time. Raungkaewmanee et al. took samples before surgery, while this study took samples after surgery.(Raungkaewmanee et al., 2012).

Some of the biochemical markers of inflammatory response have been included in prognostic scores on some cancers. As one of the systemic inflammatory markers, NLR in the peripheral has been known as an indicator of poor prognosis on various cancer types (Cedres et al., 2012). The NLR in the blood was increased in patients with advanced and progressive disease conditions. Miao et al., (2016) examined the role of NLR to predict ovarian cancer response in platinum-based chemotherapy and stated that patients with an NLR value of less than 3.02 had better progression-Free survival (PFS), with a medium PFS of 33, than the patients with an NLR over 3.02, with a medium PFS of 11 months (p < 0.001). The data from Miao et al., (2016) also in line with Badora-Rybicka et al., (2016) which assesses the neutrophil-to-lymphocyte ratio in the new patients diagnosed with ovarian cancer before chemotherapy, and found that the higher pretreatment NLR value was an adverse prognostic.However, Miao et al., (2016) and Badora-Rybicka et al., (2016) study differs from Supachai Raungkaewmanee et al., (2012), which observed that NLR did not affect PFS and ovarian cancer.

In this study, there was a significant difference between the mean NLR in the platinum-sensitive and platinum-resistant groups; a cutoff value of more than 2.56 could act as a prognostic indicator of sensitivity to platinum chemotherapy. Lastly, this study has limitations. For example, it only took blood samples after surgery; it would be better to compare the PLR and NLR values before and after surgery.

In conclusion, a patient’s NLR and PLR are strongly associated with his response to chemotherapy. NLR and PLR reflect immunity response parameters and can be obtained easily and relatively inexpensively. They are independent prognostic factors in some cancers; thus, they can help optimize decision-making during chemotherapy (or they can help achieve optimal decision-making during chemotherapy). 

## Author Contribution Statement

1. Study Design, 2. Data Collection, 3. Statistical Analysis, 4. Data Interpretation, 5. Manuscript Preparation, 6. Literature Search, 7. Funds Collection, GNAW 1,2,4,5,6,7, MP 1,2,4,5,6, HS 1,3,6, ASN 5,6, ABH 4,6, HY 4,6, DS 4,6, NT 3.

## References

[B1] Azab B, Shah N, Radbel J (2013). Pretreatment neutrophil/lymphocyte ratio is superior to platelet/lymphocyte ratio as a predictor of long-term mortality in breast cancer patients. Med Oncol.

[B2] Badora-Rybicka A, Nowara E, Starzyczny-Słota D (2016). Neutrophil-to-lymphocyte ratio and platelet-to-lymphocyte ratio before chemotherapy as potential prognostic factors in patients with newly diagnosed epithelial ovarian cancer. ESMO Open.

[B3] Cedres S, Torrejon D, Martinez A (2012). Neutrophil to lymphocyte ratio (NLR) as an indicator of poor prognosis in stage IV non-small cell lung cancer. Clin Trans Oncol.

[B4] Cho H, Hur HW, Kim SW (2009). Pre-treatment neutrophil to lymphocyte ratio is elevated in epithelial ovarian cancer and predicts survival after treatment. Cancer Immunol Immunother.

[B5] Coffelt SB, Wellenstein MD, de Visser KE (2016). Neutrophils in cancer: neutral no more. Nat Rev Cancer.

[B6] Deng F, Xu X, Lv M (2017). Age is associated with prognosis in serous ovarian carcinoma. J Ovarian Res.

[B7] DeStefano MA, Jacinto E (2013). Regulation of insulin receptor substrate-1 by mTORC2 (mammalian target of rapamycin complex 2). Biochem SocTrans.

[B8] Diem S, Schmid S, Krapf M (2017). Neutrophil-to-Lymphocyte ratio (NLR) and Platelet-to-Lymphocyte ratio (PLR) as prognostic markers in patients with non-small cell lung cancer (NSCLC) treated with nivolumab. Lung Cancer.

[B9] Edwardson DW, Boudreau J, Mapletoft J (2017). Inflammatory cytokine production in tumor cells upon chemotherapy drug exposure or upon selection for drug resistance. PLoS One.

[B10] Geng Y, Ju Y, Ren F (2014). Insulin receptor substrate 1/2 (IRS1/2) regulates Wnt/β-catenin signaling through blocking autophagic degradation of dishevelled2. J Biol Chem.

[B11] Guo Y-H, Sun H-F, Zhang Y-B (2017). The clinical use of the platelet/lymphocyte ratio and lymphocyte/monocyte ratio as prognostic predictors in colorectal cancer: a meta-analysis. Oncotarget.

[B12] Hu Q, Hada A, Han L (2020). Platelet count as a biomarker for monitoring treatment response and disease recurrence in recurrent epithelial ovarian cancer. J Ovarian Res.

[B14] Jiang Y, Qu S, Pan X (2018). Prognostic value of neutrophil-to-lymphocyte ratio and platelet-to-lymphocyte ratio in intensity modulated radiation therapy for nasopharyngeal carcinoma. Oncotarget.

[B15] Liu Y, Liu H, Luo X (2015). Overexpression of SMYD3 and matrix metalloproteinase-9 are associated with poor prognosis of patients with gastric cancer. Tumor Biol.

[B16] Martini M, De Santis MC, Braccini L (2014). PI3K/AKT signaling pathway and cancer: an updated review. Ann Med.

[B18] Mendiola M, Redondo A, Heredia-Soto V (2018). Predicting response to standard first-line treatment in high-grade serous ovarian carcinoma by angiogenesis-related genes. Anticancer Res.

[B19] Menter DG, Tucker SC, Kopetz S (2014). Platelets and cancer: a casual or causal relationship: revisited. Cancer Metast Rev.

[B20] Miao Y, Yan Q, Li S (2016). Neutrophil to lymphocyte ratio and platelet to lymphocyte ratio are predictive of chemotherapeutic response and prognosis in epithelial ovarian cancer patients treated with platinum-based chemotherapy. Cancer Biomarkers.

[B21] Neofytou K, Smyth EC, Giakoustidis A (2014). Elevated platelet to lymphocyte ratio predicts poor prognosis after hepatectomy for liver-only colorectal metastases, and it is superior to neutrophil to lymphocyte ratio as an adverse prognostic factor. Med Oncol.

[B22] Ocana A, Nieto-Jiménez C, Pandiella A (2017). Neutrophils in cancer: prognostic role and therapeutic strategies. Mol Cancer.

[B23] Olsson A-K, Cedervall J (2018). The pro-inflammatory role of platelets in cancer. Platelets.

[B24] Querleu D, Planchamp F, Chiva L (2017). European Society of Gynaecological Oncology (ESGO) guidelines for ovarian cancer surgery. Int J Gynecol Cancer.

[B25] Raungkaewmanee S, Tangjitgamol S, Manusirivithaya S (2012). Platelet to lymphocyte ratio as a prognostic factor for epithelial ovarian cancer. J Gynecol Oncol.

[B26] Savant SS, Sriramkumar S, O’Hagan HM (2018). The role of inflammation and inflammatory mediators in the development, progression, metastasis, and chemoresistance of epithelial ovarian cancer. Cancers.

[B27] Schlesinger M (2018). Role of platelets and platelet receptors in cancer metastasis. J Hematol Oncol.

[B28] Sung H, Ferlay J, Siegel RL (2021). Global cancer statistics 2020: GLOBOCAN estimates of incidence and mortality worldwide for 36 cancers in 185 countries. CA Cancer J Clin.

[B30] Zhou X, Du Y, Huang Z (2014). Prognostic value of PLR in various cancers: a meta-analysis. PLoS One.

[B31] Zuo X, Kong W, Feng L (2019). Elevated platelet distribution width predicts poor prognosis in hepatocellular carcinoma. Cancer Biomarkers.

